# Usability of the SedLine® electroencephalographic monitor of depth of anaesthesia in pigs: a pilot study

**DOI:** 10.1007/s10877-022-00807-3

**Published:** 2022-01-20

**Authors:** A. Mirra, D. Casoni, P. Barge, D. Hight, O. Levionnois, C. Spadavecchia

**Affiliations:** 1 Section of Anaesthesiology and Pain Therapy, Department of Clinical Veterinary Medicine, Vetsuisse Faculty, University of Bern, Bern, Switzerland; 2 Department for Biomedical Research, Faculty of Medicine, University of Bern, Bern, Switzerland; 3 Division of Clinical Radiology, Vetsuisse Faculty, University of Bern, Bern, Switzerland; 4 Department of Anaesthesiology and Pain Medicine, Inselspital, Bern University Hospital, University of Bern, Bern, Switzerland

**Keywords:** Pig, General anaesthesia, Sedline, Electroencephalogram, Spectrogram, Density spectral array

## Abstract

To investigate the usability of the SedLine® monitor in anaesthetized pigs. Five juvenile healthy pigs underwent balanced isoflurane-based general anaesthesia for surgical placement of a subcutaneous jugular venous port. The SedLine® was applied to continuously monitor electroencephalographic (EEG) activity and its modulation during anaesthesia. Computer tomography and magnetic resonance were performed to investigate the relationship between electrodes’ positioning and anatomical structures. The pediatric SedLine® EEG-sensor could be easily applied and SedLine®-generated variables collected. An EEG Density Spectral Array (DS) was displayed over the whole procedure. During surgery, the EEG signal was dominated by elevated power in the delta range (0.5–4 Hz), with an underlying broadband signal (where power decreased with increasing frequency). The emergence period was marked by a decrease in delta power, and a more evenly distributed power over the 4–40 Hz frequency range. From incision to end of surgery, mean SedLine®-generated values (± standard deviation) were overall stable [23.0 (± 2.8) Patient State Index (PSI), 1.0% (± 3.8%) Suppression Ratio (SR), 8.8 Hz (± 2.5 Hz) Spectral Edge Frequency 95% (SEF) left, 7.7 Hz (± 2.4 Hz) SEF right], quickly changing during emergence [75.3 (± 11.1) PSI, 0.0 (± 0.0) SR, 12.5 (± 6.6) SEF left 10.4 (± 6.6) SEF right]. Based on the imaging performed, the sensor does not record EEG signals from the same brain areas as in humans. SedLine®-DSA and -generated variables seemed to reflect variations in depth of anaesthesia in pigs. Further studies are needed to investigate this correlation, as well as to define the species-specific brain structures monitored by the EEG-sensor.

## Introduction

The porcine model is extensively used in translational medicine due to the anatomical and physiological similarities between humans and pigs [1–4]. The latest European Union’s (EU) Report on animal research estimated around 80,000 pigs used per year in the EU [5]. Despite the large number of animals involved in translational studies, objective and efficacious methods to assure suitable depth of anaesthesia (DoA) during experimental procedures are still missing. Ensuring an adequate DoA during surgical or diagnostic procedures is of paramount importance from an ethical perspective, as well as to guarantee scientific quality of collected data [6].

In order to assess DoA, physiological variables (e.g. heart rate, respiratory rate, blood pressure) as well as the presence of motor reactions to nociceptive stimulation, are generally evaluated. However, these parameters correlate more closely to modulation of the spinal cord than to depression of consciousness, which originates in the forebrain [7–9].

In order to specifically characterise and quantify brain activity during anaesthesia, several electroencephalographic (EEG) monitor devices have been developed over the last 30 years [10, 11]. Due to the complexity of raw EEG interpretation, algorithms have been patented to provide an immediate and continuous DoA index for specific use in humans. Although some of them have been used to assess DoA in pigs undergoing anaesthesia [12–14], none of them has been validated in this species so far. Moreover, their accuracy in assessing drug-induced modulation of the cerebral nervous system activity is debated, both in human and veterinary medicine. More recently, the use of the EEG spectrogram has been suggested as more appropriate for this purpose [15, 16]. Observation of the real-time spectrogram to facilitate interpretation of raw EEG signals in patients undergoing general anaesthesia could possibly contribute to improve DoA assessment in various animal species.

**Fig. 1:**
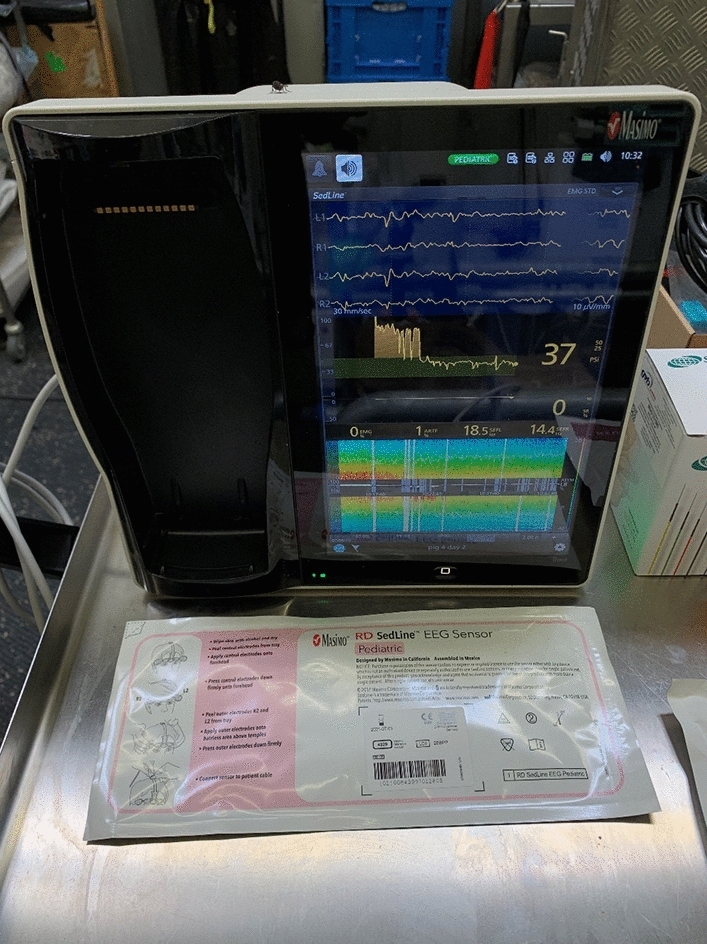
SedLine® monitor, used during general anaesthesia in pigs

The SedLine® monitor (Masimo Corp., CA, USA; see Fig. 1) displays a real-time EEG spectrogram (Density Spectral Array [DSA]) recorded from bilateral (left and right) frontal and pre-frontal (in human) transcutaneous electrodes (RD SedLine® EEG-sensor). The time course of the raw EEG is displayed and can be exported to files in the European Data Format (.edf). Moreover, five further SedLine®-generated variables are also displayed and their time course can be exported as.csv file; these are:

1. The patient state index (PSI)—processed EEG variable related to the effect of anaesthetic agents in humans, ranging between 0 (complete EEG suppression) and 100 (fully awake). Suggested target during general anaesthesia in humans is a value between 25 and 50;
2. The suppression ratio (SR, in %)—a measure of the EEG activity suppression (isoelectric signal);
3. The left and right 95% spectral edge frequencies (SEF)—a measure of the frequency below which 95% of the total EEG power is located;
4. The electromyographic activity (EMG; in %)—a measure of muscular activity contaminating the EEG signal;
5. The artifact (ARTF, in %)—a measure of physiological (not brain-related) and environmental noise contaminating the EEG signal.

Exact algorithms used to process these SedLine®-generated variables have not been made public.

To date, the use of the SedLine® monitor has not been reported in pigs. The present pilot study aimed at investigating the usability of the SedLine® monitor in anaesthetized juvenile pigs and reporting the adequacy of the SedLine® EEG-sensor for use in these patients.

## Materials and methods

A permission to perform the study was obtained from the Committee for Animal Experiments of the Canton of Bern (protocol number BE116/19).

Five healthy pigs (four males, one female; phenotypic Edelschwein), 10.4 ± 0.5 weeks old, with a mean body weight of 25.8 ± 2 kg were included. All animals underwent general anaesthesia for surgical placement of a subcutaneous jugular venous port. This was a preliminary step for a further experimental study, which is not reported here.

The animals were collected from the farm of origin the day before surgery and transported to a dedicated ward. They were housed in single boxes, but direct visual, olfactory and auditory contact with group mates was always granted. Pigs were fasted at least 6 h before surgery, while water was available ad libitum.

The day of surgery, clinical examination was performed and each pig was brought to the operation theatre where it was allowed to rest for at least 30 min in a quiet area. Ketamine (Narketan 10, Vetoquinol AG, Switzerland) 10 mg/kg, dexmedetomidine (Dexdomitor, Provet AG, Switzerland) 0.02 mg/kg and methadone (Methadon Streuli, Streuli Pharma AG, Switzerland) 0.2 mg/kg were mixed in the same syringe and injected intramuscularly in the neck. If after 15 min the sedation level was considered insufficient, a further dose of ketamine and/or dexmedetomidine and/or midazolam (Dormicum, Roche Pharma, Switzerland) was administered, as deemed necessary by the anaesthetist in charge. As soon as the animal showed signs of sedation, pre-oxygenation (4 L/min, 100% oxygen, via face mask) was initiated. A venous catheter was inserted in an auricular vein, and induction of anaesthesia performed with propofol (Propofol 1% MCT, Fresenius Kabi AG, Switzerland), administered intravenously (IV) to effect. The trachea was intubated and the endotracheal tube connected to a rebreathing system. Maintenance of anaesthesia was performed using isoflurane (Attane Isoflurane, Provet AG, Switzerland) in oxygen. Lack of palpebral reflex, jaw tone, absence of autonomic reactions and absence of reaction to external stimuli were targeted to ensure an adequate anaesthetic depth. An infusion of dexmedetomidine (0.004–0.008 mg/kg/h) was administered until full recovery. An arterial catheter was placed in either the coccygeal or the auricular artery. Continuous monitoring of heart rate, respiratory rate, invasive blood pressure, end-tidal carbon dioxide (ETCO_2_) and isoflurane (EtIso), oxygen saturation (SpO_2_), tidal volume and lung compliance was performed.

After full instrumentation, the area between the frontal and the occipital bone was clipped, cleaned with a warm antiseptic soap solution and shaved with a razor. Once dried, the skin was rubbed with abrasive paper (Red Dot Trace Prep, 3 M Health Care, Canada) and benzinum medicinale (Benzinum Medicinale, Hänseler AG, Switzerland) was applied using a soaked cotton ball to defatten the skin.

**Fig. 2:**
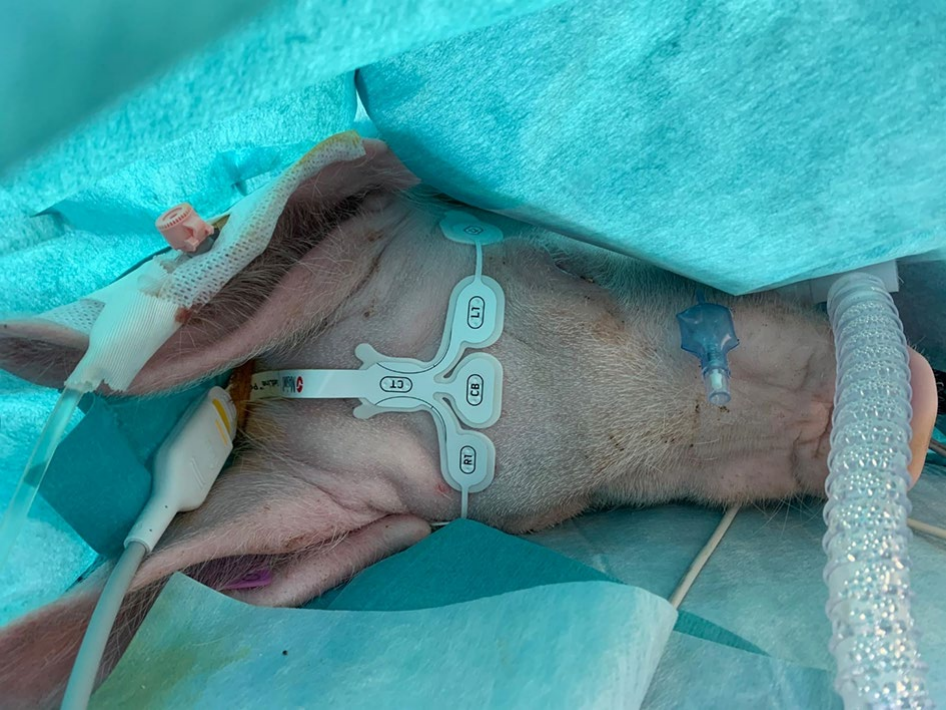
Positioning of the paediatric SedLine® electrodes in a pig. The electrodes line (L2, L1, R1, R2, from left to right) was placed over the frontal bone, between the eyes, keeping the rostral border of the electrodes on an imaginary line running between the lateral canthi of the eyes. The central GB (ground) and the caudal CT (reference) electrodes were placed on the mid-sagittal line

Once the skin was dry, the RD SedLine® EEG-sensor was positioned (adult or paediatric size). The electrodes (L2, L1, R1, R2, from left to right; see Fig. 2) were placed on a transverse line over the frontal bone, keeping their rostral border on an imaginary line running between the lateral canthi of the eyes. Left and right electrodes were placed with best possible symmetry apart the mid-sagittal line. The central CB (ground) and the caudal CT (reference) electrodes were placed on the mid-sagittal line. The adult sensor was placed first and replaced by the pediatric if judged too large (L2 and R2 too far from the temporal region, caudal to the lateral canthus of the eye). The sensor was also repositioned if the impedance was above 10 kOhm. The SedLine®-display parameters were set at 10 μV/mm and 30 mm/sec. Digital recording of the SedLine®-generated variables and raw EEG data was set. Raw EEG data were later analysed in Matlab® in order to generate the spectrogram off-line.

**Fig. 3:**
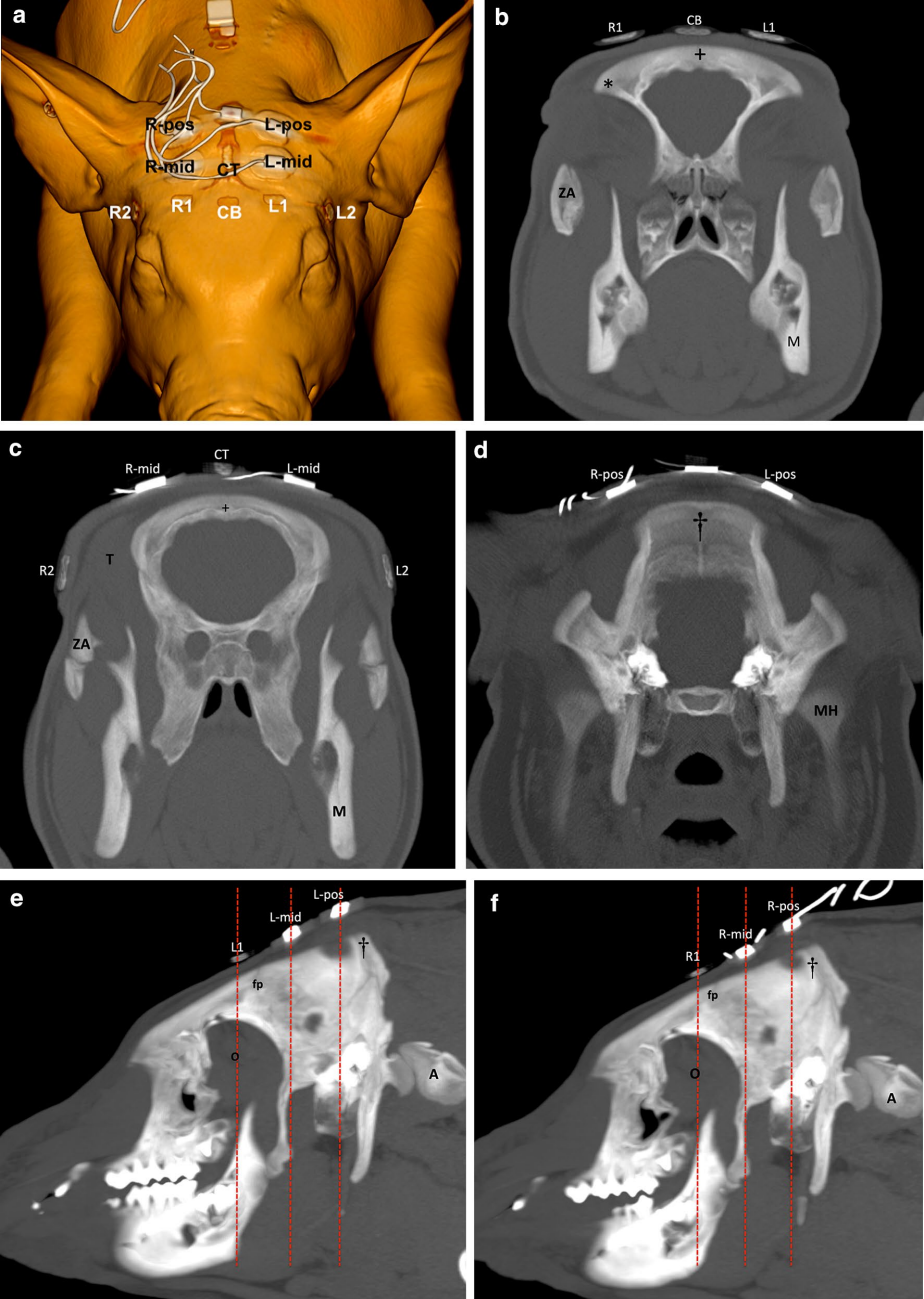
**a** Volume rendering, 3D image of a pig head, representing the positioning of the SedLine® paediatric-sensor electrodes (R2, R1, L1, L2, CB = ground, CT = reference), and further four EEG electrodes (left middle = L-mid; right middle = R-mid; left posterior = L-pos; right posterior = R-pos) positioned at an equidistance between the R1/L1 and the protuberantia occipitalis. **b** Transverse 8 mm maximum intensity projection (MIP) image demonstrating the position of the “R1” and “L1” electrodes in relation to the zygomatic processes of the frontal bone (asterisks). The “CB” electrode is positioned on the mid sagittal aspect of the skull just dorsal to the sagittal fissure (+). *ZA* Zygomatic arch, *M* mandible. Right side is on the left side of the image. **c** Transverse 8 mm MIP image demonstrating the position of the “R2” and “L2” electrodes in relation to the zygomatic arch (ZA) and temporal muscle (T). Both, the “R-mid” and “L-mid” electrodes are in the region of the parietal bone. The CB electrode is similarly positioned on the mid sagittal aspect of the skull just dorsal to the sagittal fissure (+). *M* mandible. Right side is on the left side of the image. **d** Transverse 8 mm MIP image demonstrating the position of the “R-pos” and “L-pos” electrodes in relation to the protuberantia occipitalis (dagger). *MH* mandible head. Right side is on the left side of the image. **e** Sagittal image demonstrating the position of “L1”, just rostral to the left frontoparietal suture (fp), “L-mid”, dorsal to the parietal bone, and “L-pos”, dorsal to the protuberantia occipitalis (dagger). *A* atlas, *O* orbital region. Rostral is on the left side of the image. Dashed lines indicate the region of the transverse images (Fig. 3b, c, d). **f** Sagittal image demonstrating the position of “R1”, just rostral to the left frontoparietal suture (fp), “R-mid”, dorsal to the parietal bone, and “R-pos”, dorsal to the protuberantia occipitalis (dagger). *A* atlas, *O* orbital region. Rostral is on the left side of the image. Dashed lines indicate the region of the transverse images (Fig. 3b, c, d)

Four additional EEG electrodes (not related to the SedLine® monitor) were placed more caudally. Left (L-) and right (R-) electrodes were placed symmetrically apart the mid-sagittal line, on the same sagittal line than L1 and R1, just rostral to the caudal margin of the occipital bone (L-pos, R-pos), and in the middle between these and the RD SedLine® EEG-sensor (L-mid, R-mid, see Fig. 3). These electrodes were used to collect EEG data for another investigation, which will not be reported here.

At least 10 min before starting the procedure, ropivacaine (ROPIvacain Fresenius, Fresenius Kabi, AG, Switzerland; 1 mg/kg) was injected subcutaneously at the incision site by the surgeon. Before incision, amoxicillin and clavulanic acid (Clamoxyl, GlaxoSmithKline, Switzerland; 20 mg/kg) was administered intravenously. The jugular venous catheter was placed in the left jugular vein and the subcutaneous port sutured cranial to the scapula, 2–4 cm lateral from the midline. After surgery, flunixin meglumine (Fluniximin, Graeub, Switzerland; 4.4 mg/kg) was injected intravenously. After tracheal extubation, data collection was stopped and all the monitoring tools were removed. The animal was placed in sternal recumbency in a recovery box with supplemental oxygen (4 L/min, 100%, via face mask). Sedation was administered if signs of excitation were noticed (dexmedetomidine 0.001–0.002 mg/kg IV). Once fully awake, the animal was transported back to the ward.

Two of the pigs were euthanized few days later at the end of the main study (as foreseen by the ethical authorization) and were used to perform a computed tomography and magnetic resonance imaging (MRI) of the skull, respectively. During the CT, the pediatric SedLine® sensor was left positioned as during the experiment. During the MRI, the electrodes (which are not MRI compatible) were replaced by a gadolinium-based contrast agent at the same location.

## Results

Anaesthesia was uneventful in all animals, and no further medications were administered, except one ketamine bolus (1 mg/kg IV) during the surgical procedure in the fifth pig. Surgery time was 64 ± 8.6 min (mean ± standard deviation), time from end of surgery until sternal recumbency 75 ± 23 min, end-tidal isoflurane concentration during the procedure 1.50 ± 0.15% (approximately 0.75 of the minimum alveolar concentration for pigs[17]).

In all the animals, the adult RD SedLine® EEG-sensor was judged too large, while the pediatric size was deemed more appropriate and used for all the recordings. The impedance reading was satisfactory in all animals and none of the electrodes required replacement. The sensor remained in place without the need of further fixation (bandage, glue).

The complete raw EEG (.edf) data could be retrieved for all the animals, except for pig 2, for which only a partial recording could be retrieved. The files containing SedLine®-generated variables (.csv file) revealed brief punctual loss of data, and for one pig (pig 2) the entire file was empty. No apparent reason was found for this loss of data; no artefact, electrodes or cables disconnection were noticed.

**Fig. 4:**
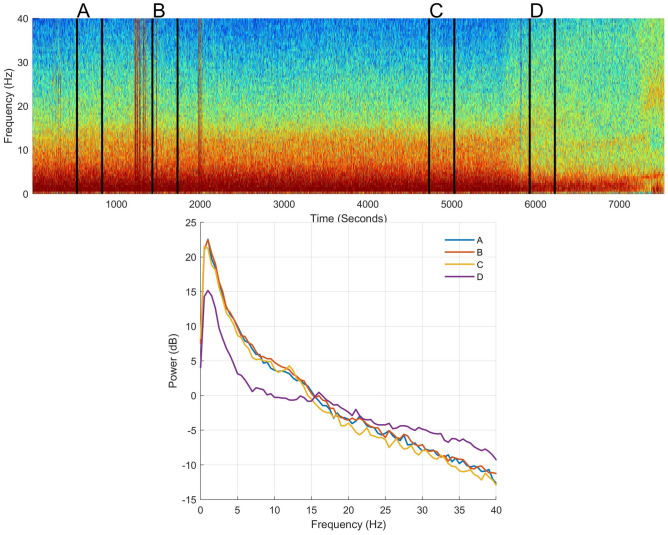
Spectrogram (top section) and power spectra of the electroencephalogram (EEG) for selected time points (bottom section) of pig 1 while undergoing general anaesthesia. EEG was taken from electrode R1, with the reference placed at CT (see Fig. 3a). The mean spectra present the power at each frequency at four different time points: A = 10 min before incision, B = 5 min after incision; C = 5 min before end of surgery; D = 15 min after the end of surgery. In case of presence of noise in the raw EEG data, data were taken from the closest noise-free EEG periods within a 3 min epoch. These time points are also shown on the spectrogram with vertical black lines and letters above, signalling the start and end time-points used to create the mean spectra

During surgery, the EEG signal was dominated by elevated power in the delta range (0.5–4 Hz), with an underlying broadband signal (where power decreased with increasing frequency). The emergence period was marked by a decrease in delta power, and a more evenly distributed power over the 4–40 Hz frequency range (Fig. 4). The EEG spectrogram (DSA) was displayed over the whole procedure for every pig. EEG power for each frequency band (Delta 0.5–4 Hz, Theta 4–8 Hz, Alpha 8–12 Hz, Beta 12–20 Hz, High Beta 20–35 Hz) at different time points is reported in Table 1.

**Table 1:** EEG power calculated at different time points during the procedure

	A	B	C	D
Delta				
Mean	14.4	17.9	16.6	13.9
SD	2.6	1.7	1.1	2.1
Theta				
Mean	6.4	8.5	6.2	2.9
SD	1.6	1.5	1.1	0.3
Alpha				
Mean	1.3	3.8	1.7	− 1.4
SD	2.1	1.5	1.7	1.3
Beta				
Mean	− 3.8	− 1.7	− 2.7	− 3.1
SD	2.5	1.5	1.3	2.1
High beta				
Mean	− 10.4	− 8.5	− 8.2	− 6.2
SD	2.9	1.7	1.1	1.6

EEG power (dB; mean and standard deviation (SD)) calculated from 4 pigs (pig 2 was excluded due to lack of data) for different frequency bands (Delta 0.5–4 Hz, Theta 4–8 Hz, Alpha 8–12 Hz, Beta 12–20 Hz, High Beta 20–35 Hz) at 4 different time points (A = 10 min before incision, B = 5 min after incision; C = 5 min before end of surgery; D = 15 min after the end of surgery). In case of presence of noise in the raw EEG data, data were taken from the closest noise-free EEG periods within a 3 min epoch

**Fig. 5:**
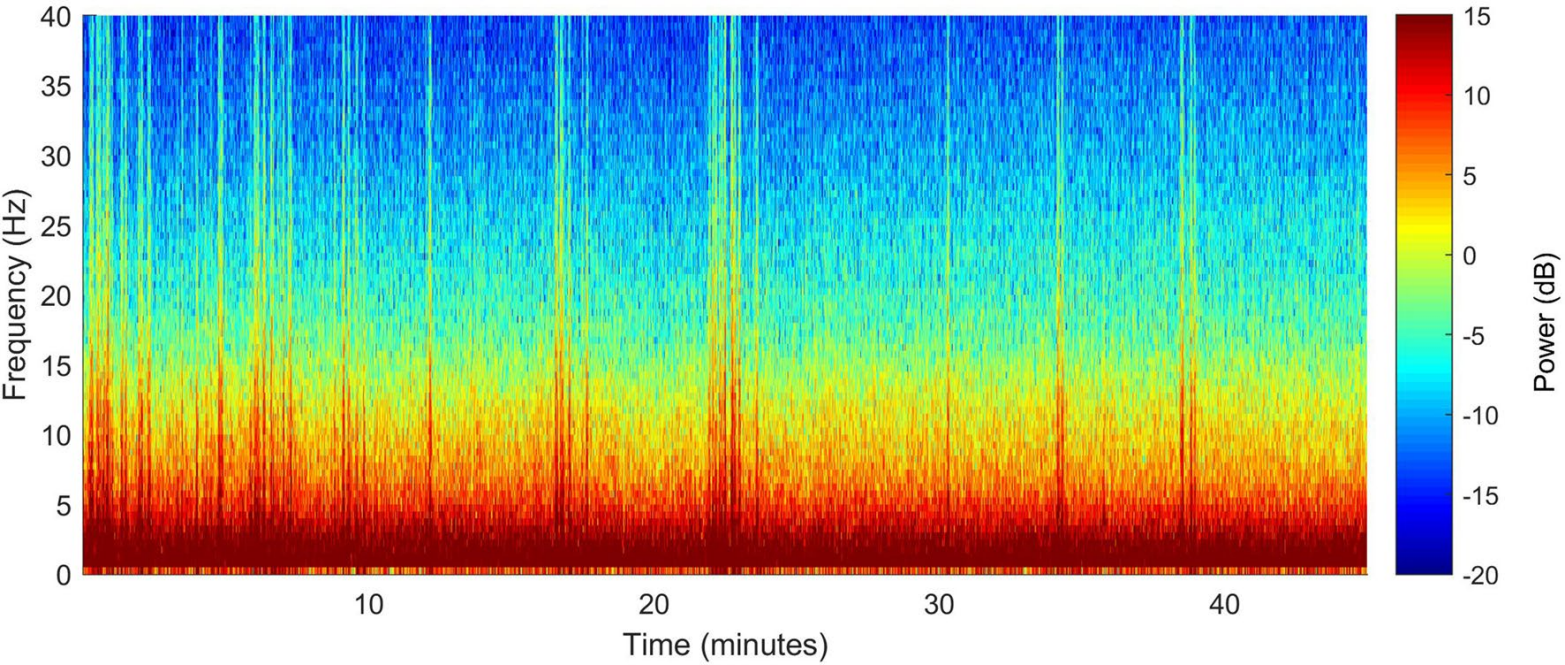
Group level (pig 2 missing because only a partial recording could be retrieved) mean spectrogram of the maintenance anaesthesia period, with time periods aligned to 5 min following incision for surgery (t = 0 min). EEG was taken from electrode R1, with the reference placed at CT (see Fig. 3a)

Sedline values were stable over the whole surgical period (from incision to end of surgery); mean values (± standard deviation) were: 23.0 (± 2.8) for PSI, 1.0% (± 3.8%) for SR, 8.8 Hz (± 2.5 Hz) for SEF l, 7.7 Hz (± 2.4 Hz) for SEF r, 3.7% (± 2.7%) for EMG, and 3.1% (± 9.6%) for ARTF. During recovery, a rapid change of the Sedline-generated variables was noticed. Values at different time points are reported in Table 2. A group level spectrogram (from 4 pigs) showing 45 min of the maintenance anesthesia period, with time periods aligned to 5 min following incision for surgery (t = 0 min) is also shown for clarity (Fig. 5).

**Table 2:** Sedline values at different time points during the procedure

	A	B	C	D
PSI				
Mean	19.4	23.0	24.2	75.3
SD	5.6	3.2	1.7	11.1
EMG				
Mean	2.2	3.0	5.0	22.9
SD	2.2	1.7	3.5	10.0
SR				
Mean	17.8	0.4	0.0	0.0
SD	24.8	1.7	0.0	0.0
SEF l				
Mean	9.5	9.0	8.5	12.5
SD	1.9	2.3	2.6	6.6
Sef r				
Mean	9.0	8.1	7.3	10.4
SD	1.9	2.1	2.6	6.6
ART				
Mean	3.7	5.9	0.1	0.0
SD	9.1	9.5	0.3	0.0

Sedline values (mean and SD) of 4 pigs (pig 2 was excluded due to lack of data; time point D from pig 5 is missing due to early removal of the EEG-sensor) at 4 different time points (A = 10 min before incision, B = 5 min after incision; C = 5 min before end of surgery; D = 15 min after the end of surgery). In case of presence of noise in the raw EEG data, data were taken from the closest noise-free EEG periods within a 3 min epoch

*PSI* Patient State Index, *EMG* electromyographic activity (%), *SR* suppression ratio (%), *SEF l* left Spectral Edge Frequency 95% left (Hz), *SEF r* right Spectral Edge Frequency 95% (Hz), *ART* artifact (%)

During electrical cautery, artifacts were visible on the monitor, and neither a clear spectrogram/raw EEG nor derived values were available in the majority of the cases.

No skin damage was visible at the sites of electrodes application after recovery.

**Fig. 6:**
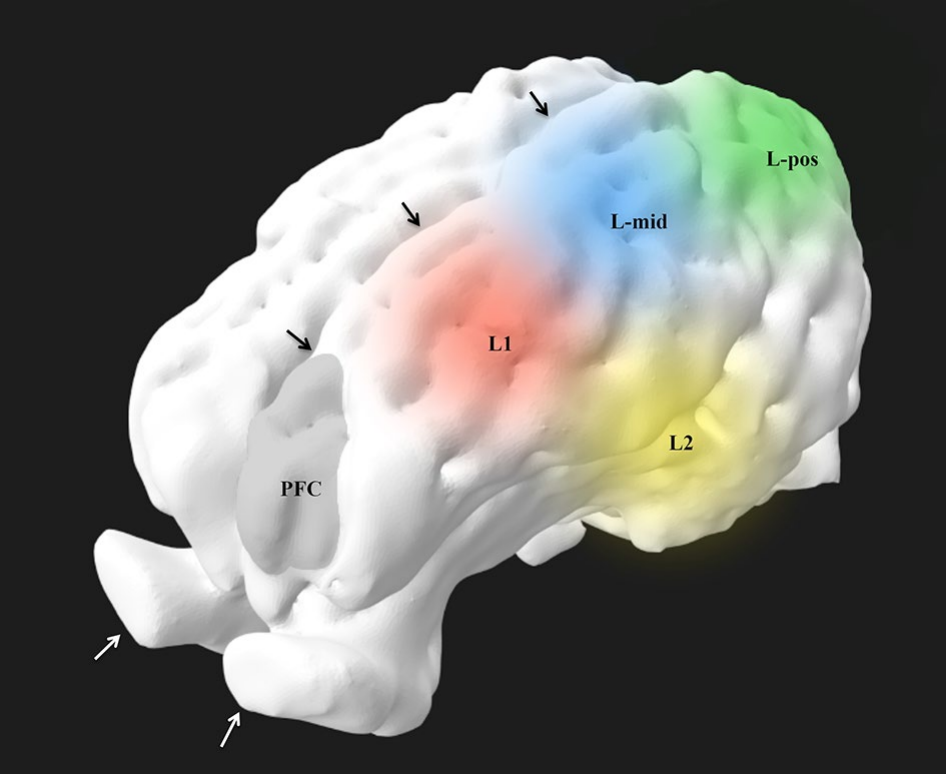
Volume rendering reconstruction of the left side of brain after skull stripping, viewed from rostral and dorsal. The round areas demonstrate the approximate brain surface coverage of the electrodes in relation with the region of the pre-frontal cortex (PFC). Black arrows: interhemispheric fissure; white arrows: olfactory bulbs

The computed tomography and MRI studies identified the anatomical regions underlying the electrodes (Figs. 3a–g, 6); details are reported in Table 3.

**Table 3:** Relationship between bone structures and brain regions/structures, identified with computed tomography (CT) and magnetic resonance (MRI) imaging in one pig

Electrodes	CT	MRI
L1	*With respect to bone structures*: dorsal and rostral the frontoparietal suture, dorsomedial to the zygomatic process of the frontal bone (around 2.5 cm)	Within the dorsolateral prefrontal cortex [24]/motor part of the frontal cortex [25, 26], approximately covering the region of the somatosensory and insular cortex [27]/temporoparietal cortex [28]
R1	As “L1”	As “L1”
L2	*With respect to bone structures*: at the level of the frontoparietal suture, lateral and slightly caudal to the zygomatic process of the frontal bone; around 1 cm dorsal to the zygomatic arch *With respect to soft tissues:* temporal fascia, rostral aspect of the left temporal muscle	Approximately at the intersection of rhinal fissure and Sylvian fissure (rostral aspect), fissures that delimit different lobes (according to Bjarkam [25]: parietal, temporal, perirhinal/insular and subrhinal lobes)
R2	As “L2”	As “L2”
CB (ground)	*With respect to bone structures*: dorsal to the sagittal suture, rostral to the frontoparietal suture	Over the interhemispheric fissure, and bilaterally over the cruciate [24]/coronal [29] fissures, dorsal to the caudal aspect of the dorsal pre-frontal cortex [24], motor cortex aspect of the frontal lobe [25, 26], frontal cortex [28] According to Clouard [30], the pre-frontal cortex is more rostrally positioned than this electrode
CT (reference)	*With respect to bone structures*: dorsal to the sagittal suture (between both parietal bones), caudal to the frontoparietal suture	Over the interhemispheric fissure, approximately covering the region of the ansate and splenial [29] or ansate/coronal [24] fissures, as well as rostral sigmoid gyrus
L-mid	*With respect to bone structures*: dorsal to the left parietal bone, caudal to the frontoparietal suture (around 1.5 cm)	Over the region of the suprasylvian [29] fissure, covering the region of the parietal cortex
R-mid	As “L-mid”	As “L-mid”
L-pos	*With respect to bone structures*: dorsal to the left parietooccipital suture, lateral to the protuberantia occipitalis around (2.5 cm)	Occipital cortex
R-pos	As “L-pos”	As “L-pos”

For visualization of the electrodes’ position, see Fig. 3a

Measurements made taking the shortest distance from the mid/central aspect of the electrode

Frontoparietal suture also called coronal suture

Parietooccipital suture also called lambdoid suture

Authors/publications used for anatomical reference are in brackets

## Discussion

The SedLine® EEG monitor was an easily applicable tool to record EEG signals in anaesthetised pigs.

Animals of 2–3 months of age were chosen, as pigs of this age are widely used in translational research due to the ease of handling. The pediatric RD SedLine® EEG-sensor appeared to fit very well to the pigs used in the present study. Sensor selection and placement may not be adequate for animals of significantly larger size. Importantly, the two sensor sizes process the EEG signal with different algorithms. As EEG dynamic modulation from general anaesthesia has been reported to differ between adult and children in humans [18], SedLine® uses a pediatric-specific signal processing engine to improve performance of the PSI. However, the exact differences in the algorithms are proprietary and not published, precluding conclusion on the impact of the sensor used.

During the procedure, single electrodes impedance remained adequate and there was no need for replacement. From author's experience with the use of RD SedLine® EEG-sensor in pigs (data not reported here), incomplete shaving is sufficient to impair signal quality, even when the skin is further prepared with soap and abrasive paper. This is in contrast with what reported by Drewnowska et al. [19], which used the RD SedLine® EEG-sensor in horses without previously shaving the hair, but applying tape bandaging to maintain a good contact over time.

Changes in the SedLine® EEG feed-speed and amplitude resolution on the display affects the data recording (Dincklage et al. [20]). Specifically, changes made to the feed-speed on the display during the recording cause a modification of the EEG sampling rate without notification. Moreover, selection of a low display resolution (e.g. 50–100 µV/mm), leads to an increased amount of zero-lines; on the other hand, a too fine resolution (1–2 µV/mm) leads to signal clipping. Therefore, it is fundamental to set the appropriate feed and resolution before starting the recording, in order to guarantee complete data acquisition if the raw EEG signal has to be assessed afterwards. In the present study, 30 mm/sec feed and 10 μV/mm amplitude were deemed appropriate.

Recordings of the numerical trends of the SedLine®-generated variables (.cvs file) was not always complete. In particular, data from the second pig were entirely missing. A human error cannot be fully excluded (i.e. involuntary change in the machine setting) but seems unlikely as the same setting was applied for further recordings in another pig on the same day and the data were correctly stored. Similar hiccup was experienced by the authors with another SedLine® device (not reported here). Thus, we suggest to record also manually the displayed values of the SedLine®-generated variables when subsequent data analysis is foreseen.

**Fig. 7:**
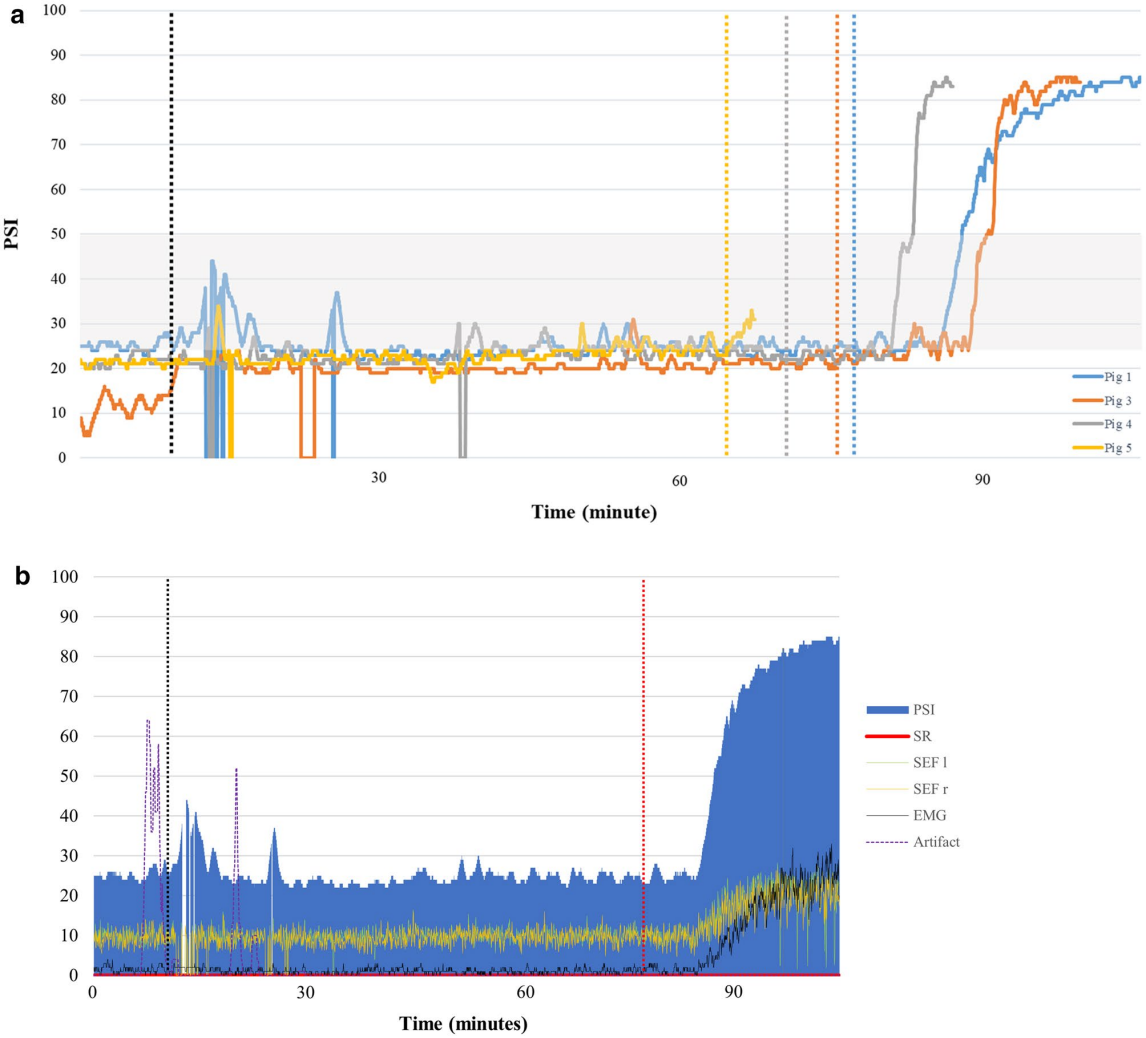
**a** PSI value for each pig (except for pig 2) is reported over time. The black vertical line shows the incision time, the other vertical lines show the end of surgery for the respective pig (see colors in the legend). The PSI values are zero when no data were recorded by the SedLine® monitor. The grey area represents the PSI range (25–50) deemed as corresponding to an adequate plane of anaesthesia in human medicine. The recovery phase in pig 5 is missing due to early removal of the EEG-sensor. **b** PSI, SR, 95% spectral edge left and right (SEF l and SEF r), EMG and artifact, recorded during the anaesthesia period in pig 1. The vertical black line represents the surgical incision, the red vertical line the end of surgery

Some small gaps in data recording were present, possibly related to external interferences (e.g. cautery usage). However, these events were not precisely recorded and this potential correlation cannot be confirmed. In any case, due to the short duration of these gaps, the overall data collection quality was not compromised (Fig. 7a, b).

No data are available yet to suggest for each SedLine®-pediatric-generated variables which target is indicative of an adequate DoA in pigs. In human medicine, a PSI between 25 and 50 is considered an optimal hypnotic state during general anaesthesia [21, 22]. In our pigs, the PSI value was always below 50 and often below 30, when clinical evaluation of DoA was deemed adequate. During the recovery phase, PSI increased quickly (mean ± SD: 75.3 ± 11.1, fifteen minutes after end of anaesthesia) reflecting the clinical picture. However, further studies are needed to investigate if SedLine®-generated variables correlate with DoA in pigs.

The report of the anatomical structures covered by the electrodes is a first step toward a better understanding of the correlation between EEG signal, skull landmarks and brain areas. Since no data are present in pigs, our results initiate refinement and standardization of electrodes positioning in this species. In humans, the SedLine® electrodes cover the regions defined as Fp1–Fp2 (prefrontal) and F7–F8 (frontal) in the 10–20 system of EEG electrodes placement [23]. Based on the computed tomography and MRI studies presented, the electrode placement applied here in pigs does not cover the same brain areas. How much these differences affect the interpretation of the EEG and the SedLine®-derived variables is not known. Precise and homogeneous information on the functional areas in the pig’s brain is still missing [25–30]. Further anatomical studies on a larger sample of animals are required.

In humans, anteriorization of the alpha waves occurs with deepening of the anaesthetic level [15, 16], justifying the relevance of monitoring EEG activity from the frontal brain lobes. No evidence of this phenomenon has been provided in pigs so far. The computed tomography and MRI studies suggest that the L1 and R1 electrodes, as placed in the present study, monitor rather the mid-frontal area, and that electrodes should potentially be placed more rostrally (on a line between the center of the eyes) to investigate EEG activity from most frontal brain areas. However, placing the sensor in a more rostral position would mean to possibly record over the frontal sinus, which is air filled. Moreover, the rigidity of the SedLine® sensors and the anatomy of the pig’s skull, would make this positioning really difficult in pigs of small size.

This paper reports the usage of the SedLine® monitor in pigs undergoing general anaesthesia. With our preparation and settings, a stable EEG signal, spectrogram and derived data were shown during the entire procedure. Artefacts can influence data recording, and data saving errors from the monitor can occur. Further studies are needed to investigate the correlation between Sedline®-generated variables and actual depth of anaesthesia in pigs.

## Data Availability

Not applicable.
